# Diagnostic efficacy of ultrasound combined with fine-needle aspiration biopsy for papillary thyroid carcinoma based on C-TIRADS and ATA guidelines

**DOI:** 10.3389/fonc.2026.1815675

**Published:** 2026-05-29

**Authors:** Zhi Zhang, Dou Wu, Yilun Wu, Jingjing Gan, Jianjian Liu

**Affiliations:** 1 Department of Ultrasound Medicine, Shanghai Public Health Clinical Center, Fudan University, Shanghai, China; 2 Health Examination Management Center, Shanghai Public Health Clinical Center, Fudan University, Shanghai, China

**Keywords:** ATA guidelines, C-TIRADS, diagnostic efficacy, fine-needle aspiration biopsy, papillary thyroid carcinoma, ultrasound

## Abstract

**Objective:**

This study aimed to analyze the diagnostic efficacy of color Doppler ultrasound combined with ultrasound-guided fine-needle aspiration biopsy (US-FNAB) for papillary thyroid carcinoma (PTC) based on the Chinese Thyroid Imaging Reporting and Data System (C-TIRADS) and the guidelines of the American Thyroid Association (ATA).

**Methods:**

A total of 478 patients were enrolled from January 2021 to December 2025, with postoperative pathological results as the gold standard. Chi-square test and binary logistic regression were used to identify risk factors. ROC curve analysis was used for diagnostic efficacy, and Kappa test was used separately for consistency. The performance of C-TIRADS and ATA was compared using equivalent malignancy risk thresholds.

**Results:**

Logistic regression showed that unclear border, irregular shape, vertical growth, microcalcification, aspect ratio >1, capsular invasion, and lobulated lesions were independent risk factors for PTC (p<0.05). Combined diagnosis achieved higher sensitivity, accuracy, and discriminative power than either single examination. By ROC analysis, the AUC of ultrasound, US-FNAB, and their combination was 0.709, 0.708, and 0.762, respectively. Kappa values showed moderate agreement with pathology. The AUC of C-TIRADS and ATA was 0.708 and 0.669, respectively. C-TIRADS showed significantly higher sensitivity, specificity and accuracy than ATA guidelines (p<0.05). Inter-observer agreement for C-TIRADS and ATA classification was good (κ=0.81, κ=0.77, respectively).

**Conclusion:**

Ultrasound combined with US-FNAB yields higher clinical value for PTC diagnosis, especially in indeterminate and discordant cases. C-TIRADS is recommended for routine risk stratification before FNAB in Chinese clinical settings.

## 1 Introduction

Papillary thyroid carcinoma (PTC) is the most common endocrine malignancy in adults. Color Doppler ultrasound detects 30–70% of thyroid nodules, 15% of which are malignant. US-FNAB is a standard tool with high diagnostic specificity (1–3). While comparisons between C-TIRADS and ATA have been reported, few studies focus on challenging nodules and discordant results between imaging and cytology. This study evaluates the diagnostic value of ultrasound combined with US-FNAB for PTC, with head-to-head comparison between C-TIRADS and ATA guidelines.

## 2 Materials and methods

### 2.1 Study subjects

478 patients admitted from January 2021 to December 2025 were enrolled. All patients underwent preoperative US-FNAB. Patients were divided into malignant (201) and benign (277) groups by postoperative pathology, including 332 females and 146 males.

#### 2.1.1 Inclusion criteria

① Thyroid nodules detected by ultrasound; ② Complete clinical and imaging data.

#### 2.1.2 Exclusion criteria

① Recent neck surgery or hemorrhage; ② Mental disorders; ③ Diffuse thyroid diseases.

All nodules were confirmed by postoperative pathology; informed consent was obtained. This study was approved by the Ethics Committee of Shanghai Public Health Clinical Center.

### 2.2 Instruments and methods

#### 2.2.1 Ultrasound equipment

SIEMENS ACUSON Sequoia Silver convex array probe, probe frequency: 3.5 MHz. The patient lies on his back with his head in a relaxed position, and the thyroid area is examined. The following features were recorded: border (clear/unclear), shape (regular/irregular), vertical growth, microcalcification, aspect ratio (>1 or ≤1), capsular invasion, lobulated lesions, lateral acoustic shadow, internal blood flow.

US-FNAB was performed under real-time guidance. Local anesthesia was administered. A 23G needle was used; 2–3 passes were performed for each nodule to ensure sample adequacy. Smears were prepared and stained with Papanicolaou or H&E. Cytological results were classified according to the Bethesda System for Reporting Thyroid Cytopathology (BSRTC). Samples were defined as adequate if containing sufficient follicular cells for diagnosis. Pathologists were blinded to ultrasound results and clinical information.

Ultrasound images were retrospectively reviewed by three senior radiologists blinded to pathology. Nodules were classified according to 2020 C-TIRADS and 2015 ATA guidelines. For consistent comparison, C-TIRADS ≥4a was defined as positive, and ATA low/moderate/high suspicion was defined as positive. Inter-observer agreement was assessed using Kappa test.

### 2.3 Observation indicators

Postoperative pathology was the gold standard. Sensitivity, specificity, accuracy, positive predictive value, negative predictive value, and AUC were calculated. Kappa test evaluated consistency between index tests and pathology. Ultrasound features were documented. Nodules were classified by C-TIRADS and ATA guidelines.

### 2.4 Statistical methods

SPSS 26.0 was used. Chi-square test was used for univariate analysis. Binary logistic regression identified independent risk factors. ROC curve analysis was performed to calculate AUC for diagnostic discrimination. Kappa test was used separately for consistency. P<0.05 was considered significant. The sample size was determined based on a prevalence of malignant nodules ~40% and a desired precision of 5%, requiring at least 400 cases; 478 cases were included to ensure reliability.

## 3 Results

### 3.1 Analysis of color ultrasonic image features of nodules in two groups

**Figure 1:**
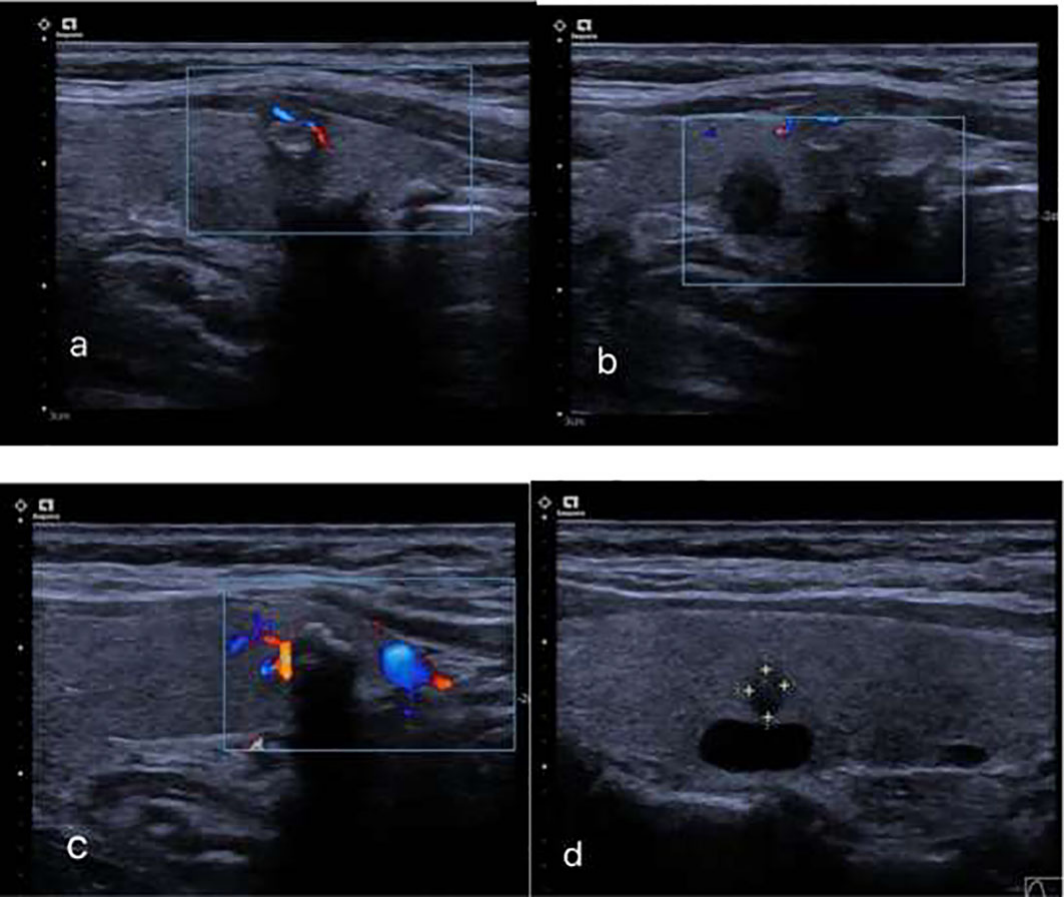
A 43-year-old male patient with multiple PTC in bilateral lobes of the thyroid gland. **(a)**. Unclear border, irregular shape, vertical growth, capsular invasion, microcalcification, aspect ratio >1, and arterial blood flow signals were seen on CDFI; **(b)**. Unclear border, irregular shape, vertical growth, capsular invasion, aspect ratio >1, and lateral echo attenuation; **(c)**. Unclear border, irregular shape, lobulated lesions, vertical growth, microcalcification, aspect ratio >1, lateral and posterior echo attenuation, and arterial blood flow signals were seen around the lesion on CDFI; **(d)**. Irregular shape, vertical growth, aspect ratio >1, and microcalcification.

Comparison between PTC (n=201) and benign nodules (n=277) showed significant differences in border, shape, vertical growth, microcalcification, aspect ratio >1, capsular invasion, lobulated lesions, lateral shadow, internal blood flow (p<0.05) (**Table 1**, **Figure 1**).

**Table 1:** Comparison of ultrasonic image features between malignant PTC nodule group and benign BTN nodule group.

Item	Subitem	PTC nodules (n=201)	BTN nodules (n=277)	χ²	P
Location	Left lobe	105	130	1.313	0.252
	Right lobe	96	147		
Gender	Female	144	188	0.781	0.377
	Male	57	89		
Border	Unclear	188	58	245.742	0.000
	Clear	13	219		
Shape	Irregular	156	96	86.222	0.000
	Regular	45	181		
Aspect ratio	>1	139	70	91.161	0.000
	≤1	62	207		
Microcalcification	Present	145	104	55.857	0.000
	Absent	56	173		
Vertical growth	Present	144	79	87.027	0.000
	Absent	57	198		
Capsular invasion	Present	58	25	31.922	0.000
	Absent	143	252		
Lateral acoustic shadow	Present	48	13	38.519	0.000
	Absent	153	264		
Lobulated lesions	Present	77	12	88.740	0.000
	Absent	124	265		
Internal blood flow	Present	57	41	13.134	0.000
	Absent	144	236		

### 2.2 Logistic regression analysis of ultrasonic image features of PTC nodules

Logistic regression analysis results showed that unclear border, irregular shape, vertical growth, microcalcification, aspect ratio >1, capsular invasion, and lobulated lesions were independent risk factors for PTC nodules (p<0.05), with odds ratios (OR) of 5.414, 3.612, 3.270, 3.366, 2.667, 2.008, and 2.261, respectively. Unclear border, irregular shape, vertical growth, microcalcification, aspect ratio >1, capsular invasion, lobulated lesions were independent risk factors (p<0.05) (**Table 2**).

**Table 2:** Binary logistic regression analysis of ultrasonic features of PTC.

Image feature	B	Standard error	Wald	Significance	Exp(B)	95%CI (lower)	95%CI (upper)
Unclear border	1.689	0.300	31.594	0.000	5.414	3.004	9.756
Irregular shape	1.284	0.301	18.226	0.000	3.612	2.003	6.514
Vertical growth	1.185	0.292	16.452	0.000	3.270	1.845	5.796
Microcalcification	1.214	0.282	18.463	0.000	3.366	1.935	5.856
Aspect ratio >1	0.981	0.292	11.313	0.001	2.667	1.506	4.725
Capsular invasion	0.697	0.344	4.106	0.043	2.008	1.023	3.942
Lateral acoustic shadow	0.541	0.448	1.455	0.228	1.717	0.713	4.133
Lobulated lesions	0.816	0.414	3.890	0.049	2.261	1.005	5.088
Internal blood flow	0.304	0.341	0.795	0.373	1.355	0.693	2.647

### 2.3 Diagnostic efficacy of ultrasound, US-FNAB, and their combination

**Figure 2:**
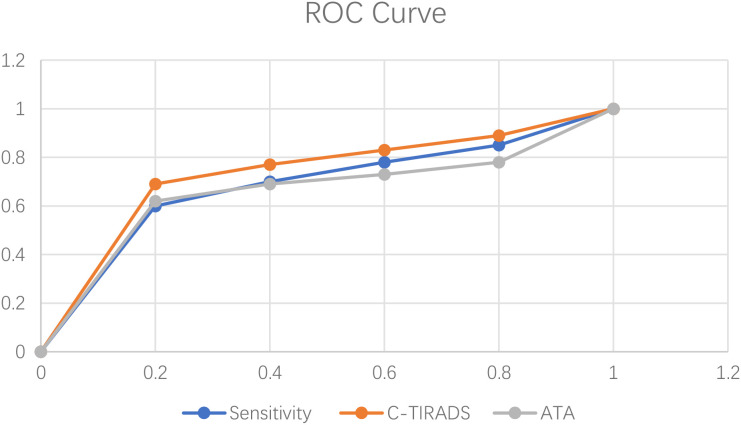
ROC curves of ultrasound, US-FNAB and their combination in differentiating benign and malignant thyroid nodules.

As shown in **Table 3** and **Figure 2**, compared with single ultrasound (AUC: 0.709) and US-FNAB (AUC: 0.708), the combined modality achieved the highest AUC of 0.762, with sensitivity of 87.40%, specificity of 83.00%, and accuracy of 89.50%. This indicates that the combined strategy significantly improves diagnostic accuracy by reducing false positive and false negative cases. Consistent with pathological results, the combined diagnosis also showed the highest Kappa value (0.80), suggesting excellent consistency (**Table 3**, **Figure 2**).

**Table 3:** Analysis of diagnostic efficacy of color doppler ultrasound, US-FNAB, and their combination.

Examination method	TP	TN	FP	FN	AUC	Sensitivity	Specificity	Accuracy
Color Doppler ultrasound	144	223	54	57	0.709	0.716	0.805	0.767
Fine-needle aspiration biopsy	156	218	59	45	0.708	0.776	0.787	0.782
Combined diagnosis	176	230	47	25	0.762	0.874	0.830	0.895

TP, represents true positive (pathologically confirmed malignant cases correctly identified by the test); TN, represents true negative (pathologically confirmed benign cases correctly identified as negative); FP, represents false positive (benign cases misclassified as malignant); FN, represents false negative (malignant cases misclassified as benign).

### 2.4 Diagnostic efficacy of C-TIRADS and ATA guidelines

**Figure 3:**
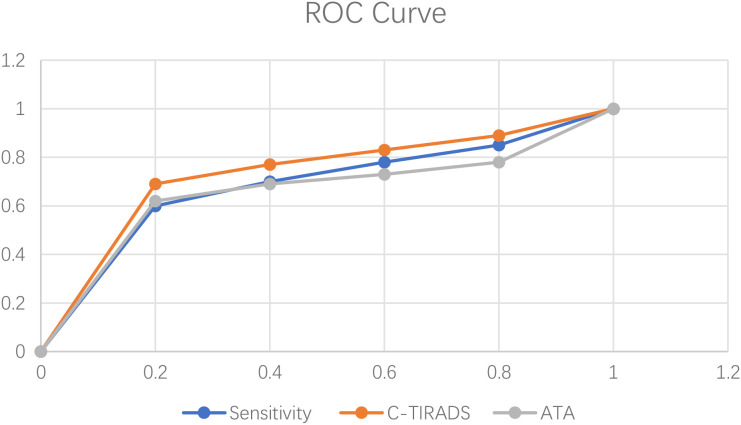
ROC curves comparing diagnostic efficacy of C-TIRADS and ATA guidelines.

C-TIRADS ≥4a and ATA low–high suspicion were used as equivalent positive thresholds. AUC: C-TIRADS 0.708 vs ATA 0.669. C-TIRADS showed higher sensitivity (0.805 vs 0.621), specificity (0.765 vs 0.650), accuracy (0.786 vs 0.680) (p<0.05) (**Table 4**, **Figure 3**).

**Table 4:** Analysis of diagnostic efficacy of C-TIRADS and ATA guidelines for thyroid nodules.

Guideline	Positive threshold	AUC	Sensitivity	Specificity	Accuracy
C-TIRADS	≥4a	0.708	0.805	0.765	0.786
ATA	Low–high suspicion	0.669	0.621	0.650	0.680

## 3 Discussion

Papillary thyroid carcinoma (PTC) is the most prevalent subtype of differentiated thyroid carcinoma, with a steadily rising global incidence. High-frequency color Doppler ultrasound has become the first-line imaging modality for thyroid nodule evaluation, detecting thyroid nodules in up to 67% of adults. Most PTC patients exhibit favorable outcomes, with a 5-year survival rate of approximately 98% (1, 2). Nevertheless, accurate preoperative risk stratification remains essential to avoid overtreatment of benign lesions and undertreatment of clinically significant malignancies. At present, ultrasound-guided fine-needle aspiration biopsy (US-FNAB) constitutes the cornerstone for evaluating the malignant risk of thyroid nodules; false-negative results at this stage may lead to delayed intervention and adversely affect prognosis. Risk stratification systems including C-TIRADS and ATA guidelines demonstrate high reliability in predicting nodule malignancy. The combined application of imaging assessment, cytological examination, and standardized classification systems can effectively improve the diagnostic accuracy of thyroid cancer, provide robust evidence for surgical planning, and support the rational use of targeted therapies (4).

In the present study, 478 patients with thyroid nodules were enrolled, with postoperative pathological findings serving as the diagnostic gold standard. All subjects were dichotomized into benign and malignant groups according to histopathological results. Chi-square test and binary logistic regression were applied to investigate the association between grayscale ultrasound features and nodule nature. Logistic regression analysis revealed that unclear border, irregular shape, vertical growth, microcalcification, aspect ratio >1, capsular invasion, and lobulated contour were independent risk factors for PTC (P<0.05). These seven high-risk ultrasound features are consistent with recent large-sample clinical investigations and meta-analyzes, confirming the reproducibility of core imaging predictors for PTC. Our findings further validate that ultrasound combined with US-FNAB yields improved diagnostic performance for PTC, particularly for cytologically indeterminate or sonographically discordant nodules.

US-FNAB exhibits favorable sensitivity and diagnostic accuracy in characterizing thyroid nodules, representing a minimally invasive, convenient, and reliable adjunctive tool for preoperative evaluation. In recent years, US-FNAB has been widely implemented in Chinese medical centers, accompanied by increasing evidence supporting its clinical value (2, 3). Color Doppler ultrasound combined with US-FNAB has been verified as an effective strategy for preoperative diagnosis of PTC. Furthermore, the integration of molecular markers such as BRAF V600E into FNA specimens has refined diagnostic precision and facilitated personalized treatment selection (5). In the current study, Kappa analysis and receiver operating characteristic curve analysis demonstrated that the AUC values of ultrasound, US-FNAB, and their combined application were 0.709, 0.708, and 0.762, respectively. According to the Kappa diagnostic criteria, values above 0.75 indicate good consistency, 0.40-0.75 moderate consistency, and below 0.40 poor consistency. Our results indicate moderate diagnostic consistency for standalone ultrasound or US-FNAB, whereas their combination achieves improved diagnostic efficacy (6, 7). However, US-FNAB has inherent limitations, including interobserver variability in cytological interpretation, variability in puncture technique, and potential procedure-related complications. Therefore, strict adherence to standardized biopsy protocols, rigorous monitoring of adverse events, and systematic training are warranted to enhance procedural safety and diagnostic accuracy.

Globally, thyroid nodule risk stratification is guided by national and international clinical guidelines. The Chinese Thyroid Imaging Reporting and Data System (C-TIRADS) was proposed by Chinese experts in 2020. Based on international consensus while adapting to domestic clinical practice, C-TIRADS adopts a stepwise approach to malignant risk stratification and provides a logically hierarchical diagnostic framework. With concise classification criteria, C-TIRADS facilitates standardized ultrasound reporting, guides subsequent contrast-enhanced ultrasound and FNAB procedures, and supports clinical decision-making. In contrast, the ATA guidelines developed by the American Thyroid Association differ in sonographic feature weighting. Notably, C-TIRADS provides more comprehensive definitions for microcalcification and lobulated lesions and offers more intuitive and clinically applicable malignant risk probabilities. In the present study, direct comparison showed that C-TIRADS achieved superior diagnostic performance relative to ATA guidelines, which may be attributed to its more inclusive sonographic criteria and clearer risk stratification optimized for the Chinese population. Internal vascularity was statistically significant in univariate analysis but not in multivariate regression, suggesting that internal blood flow may act as a confounding factor rather than an independent predictive marker for PTC.

A key objective of this study was to compare C-TIRADS and ATA guidelines. Our results demonstrated that C-TIRADS was superior to ATA in all diagnostic indicators, including AUC, sensitivity, specificity, and accuracy. Several factors may explain this advantage (8, 9). First, C-TIRADS provides a more explicit, point-based scoring system with clear definitions for microcalcification, lobulated margins, and vertical orientation. Second, C-TIRADS was developed and validated using Asian populations, making it more suitable for Chinese patients than the ATA system, which is based primarily on Western cohorts. Third, the risk stratification of C-TIRADS is more intuitive and clinically practical, reducing inter-observer variability. Although the two systems use different classification thresholds, our comparison reflects real clinical practice and provides robust evidence for prioritizing C-TIRADS in Chinese clinical settings.

This study has several limitations. First, it is a retrospective single-center design, which may lead to selection bias and limit generalizability. Second, molecular markers such as BRAF V600E were not included, which may further improve the diagnosis of indeterminate nodules. Third, subgroup analyzes based on nodule size, cystic components, or lymph node metastasis were not performed. Fourth, although inter-observer agreement was assessed, multi-center prospective studies are needed to confirm long-term stability. Despite these limitations, the large sample size and standardized procedures enhance the validity of our conclusions.

## 4 Conclusion

Conventional ultrasound provides valuable risk stratification for PTC, and ultrasound combined with US-FNAB significantly improves diagnostic accuracy compared with single examinations. C-TIRADS exhibits better diagnostic performance than the ATA guidelines in Chinese patients. Given the retrospective and single-center nature of this study, C-TIRADS is suggested as a preferred tool for preoperative risk stratification before FNAB; multi-center prospective studies are encouraged to confirm its generalizability.

## Data Availability

The original contributions presented in the study are included in the article/supplementary material. Further inquiries can be directed to the corresponding author.
